# Promoting intestinal IgA production in mice by oral administration with anthocyanins

**DOI:** 10.3389/fimmu.2022.826597

**Published:** 2022-07-27

**Authors:** Xuerun Liu, Luoyang Wang, Huiren Zhuang, Zhenghuan Yang, Guoqiang Jiang, Zheng Liu

**Affiliations:** ^1^ Key Lab of Industrial Biocatalysis Ministry of Education, Tsinghua University, Beijing, China; ^2^ Department of Chemical Engineering, Tsinghua University, Beijing, China; ^3^ School of Basic Medicine, Qingdao University, Qingdao, China

**Keywords:** gut microbiota, intestinal mucosal immune system, bilberry anthocyanins, secretory immunoglobulin A, reactive oxygen species

## Abstract

While recent years have witnessed ever-growing evidence on the prebiotic attributes of anthocyanins for treatment of microbiota-associated diseases, the complex interplay between anthocyanin uptake, the gut microbiota, and the intestinal mucosal immune system remains poorly understood. Here, we investigate the effects of bilberry anthocyanins on the gut microbiota composition and metabolism, and the intestinal mucosal immune system of mice. We observed an increased proportion of IgA-producing plasma cells in the mesenteric lymph nodes (MLNs) and an enhanced secretion of secretory immunoglobulin A (sIgA) and antimicrobial peptides in the small intestine. Small intestine transcriptome analysis further suggested that anthocyanins influenced IgA production. We found that oral administration of anthocyanins altered the gut microbiota through maintaining the anaerobic intestinal environment, promoting the secretion of sIgA and antimicrobial peptides, and downregulating cell motility and mobile genetic elements of commensal bacteria. These observations suggest that the oral administration of anthocyanins helps in maintaining intestinal homeostasis and thus it may find applications in immunotherapy and related fields.

## Introduction

Anthocyanins are water-soluble flavonoid pigments that are extensively found in fruits and vegetables such as purple sweet potato, berries, and grapes. The naturally occurring chemicals have proven health benefits including anti-inflammation (1), prevention and treatment of several chronic diseases, such as diabetes and cardiovascular disorders (2). Considerable amounts of anthocyanins may reach the colon and metabolized by gut microbiota (3, 4), resulting in the production of new phenolic compounds that can be absorbed and exert health effects (5–7). In addition, anthocyanins and their metabolites can modulate the composition and function of gut microbiota (6, 8, 9). Therefore, it is commonly hypothesized that gut microbiota may be a key factor for the desired physiological function of anthocyanins (6). It is observed that anthocyanins and their metabolites could increase the diversity of the gut microbiota, promote the growth of several beneficial bacterial groups (6), enhance the production of fecal short-chain fatty acids (SCFAs) (9, 10) and maintain the integrity of the intestinal barrier (9). Our previous studies showed that anthocyanins could induce positive changes in the composition of gut microbiota and improve the therapeutic efficiency of PD-L1 blockade treatment (11, 12). Given aforenoted efforts, our understanding of the mechanism responsible for the gut microbiota modulating activity of anthocyanins is far from adequate.

The gut microbiota is critical to maintain the host health and physiology (13). Dysbiosis of the gut microbiota is associated with several diseases including inflammatory bowel diseases (IBDs), irritable bowel syndrome (IBS), metabolic syndrome, colon cancer, and obesity (14). On the other hand, growing efforts are directed at modulating gut microbiota in order to ameliorate various commensal bacteria-associated diseases such as *Clostridium difficile* infection (CDI), IBDs and IBS (15). As an important immunological defense of the host, sIgA plays a vital role in preventing pathogen infection and maintaining the homeostasis of the gut microbiota. Passive immunization with sIgA provides a promising strategy for the prevention of enteric pathogen infection (16). Interestingly it has been reported that anthocyanins could obviously promote the intestinal IgA secretion in animal studies (17, 18), providing a novel approach for preventing gastrointestinal infections. However, the underlying mechanisms for the promotion of sIgA secretion by anthocyanins have not been fully understood.

To illustrate the interplay of anthocyanins with the gut microbiota and the intestinal mucosal immune system, herein we performed two-week anthocyanin dietary intervention in C57BL/6 male mice. We observed that the anthocyanin administration resulted in a significantly increased secretion of sIgA and antimicrobial peptides in the small intestine. Accordingly, the proportion of IgA-producing plasma cells is increased in the MLNs, accompanied by a reduction of the fecal redox potential and downregulation of reactive oxygen species (ROS) levels in the gut. PICRUSt and BugBase analyses based on 16S rRNA sequencing data suggested an enhanced proportion of anaerobic bacteria and a downregulation of cell motility and mobile genetic elements. In addition, we performed weighted gene co-expression network analysis (WGCNA) to identify specific bacterial taxa that are associated with the secretion of IgA and antimicrobial peptides. The experimental findings of the present study enhance understanding the mechanisms of anthocyanin in regulating gut microbiota and, consequentially, the intestinal immune response. The experimental results shed new light on the display of anthocyanins’ healthy attributes and function.

## Materials and methods

### Chemicals and antibodies

Standardized bilberry extract (MIRTOSELECT^®^) was purchased from Indena S.p.A. (Milan, Italy) containing 36% of anthocyanins. We had previously determined the anthocyanin composition by high-performance liquid chromatography–ultraviolet-visible spectroscopy-tandem mass spectrometry method (11). ELISA kits for immunoglobulin G (IgG), immunoglobulin M (IgM) and IgA were purchased from Invitrogen, the catalog numbers of them are respectively 88-50400-86, 88-50470-86, and 88-50450-86. ELISA kits for transforming growth factor (TGF)-β1, B-cell activating factor (BAFF), and Matrix Metallopeptidase-2 (MMP-2) were purchased from Abbkine Scientific Co., Ltd. (Wuhan, China), the catalog numbers of them are respectively KET7014, KTE71416, KTE71004. ELISA kit for LPS was purchased from Nanjing Jiancheng Bioengineering Institute (Nanjing, China), the catalog numbers is H255. Fluorophore-conjugated commercial antibodies to CD45.2 (clone: 104), CD45R (clone: RA3-6B2), IgA (clone: mA-6E1) were purchased from eBioscience and listed in **Supplementary Table S1**.

### Animals and treatment

Male C57BL/6 mice (seven weeks old) were purchased from the Beijing Vital River Laboratory Animal Technology Co. Ltd. (Beijing, China). During the experimental session, all mice were housed under specific pathogen-free conditions in the animal care facilities at the Laboratory Animal Research Center, Tsinghua University. After a one-week adaptation, mice were randomized by body weight into two experimental groups of 12 animals. For the anthocyanin group, mice were given a dose of 50 mg bilberry anthocyanin extracts/kg body weight every day for 14 days, and the day of the first intragastric administration was recorded as day 0. For the control group, mice were given isovolumetric sterile water as the anthocyanin group. The body weight of each mouse was recorded every other day. All mice were sacrificed by cervical dislocation under anesthesia with Avertin after two-week anthocyanin dietary intervention, then small intestine and colon tissues, cecal contents were collected and stored at −80°C for the following experiments. DNA extraction and bacterial identification of cecal content samples were performed as we previously described (11). All animal procedures were performed in accordance with guidelines of the Animal Care and Use Committee of Tsinghua University (No. 20-LZ1#).

### ELISA assay for immunoglobulin, cytokine, MMP-2 and LPS levels

The concentrations of serum LPS, IgG, IgM and IgA were determined by ELISA kits according to the manufacturer’s instructions.

The LPS and IgA levels in feces were assessed using the mouse ELISA kits according to the manufacturer’s instructions. Briefly, 0.1 g of feces samples were suspended in 1 mL of ice-cooled phosphate-buffered saline (PBS), and then homogenized by beat beating using a Scientz-48 grinder. After centrifugation (10000 × g, 4°C, 10 min), the supernatant was collected and further diluted for ELISA quantification of LPS and IgA contents.

For intestine tissues, approximately 0.1 g of frozen colon or small intestine tissue samples were homogenized in 1 mL of ice-cooled PBS with a Scientz-48 tissue grinder. After centrifugation (3000 × g, 4°C, 10 min), the supernatant was collected for the BCA protein assay and for ELISA quantification of sIgA, IL-6, TNF-α, TGF-β1, BAFF and MMP-2 contents, and their levels were described as the amount contained per gram protein.

### Analysis of IgA^+^ and IgA^-^ bacteria

Flow cytometric analysis of IgA-coated bacteria of feces samples was performed as Noah W. Palm et al. previously described with some modification (19). In brief, 2-3 grain of feces were suspended in 1 mL PBS, homogenized, and then centrifuged (50 × g, 15 min, 4°C) to remove larger particles. Supernatant was centrifuged at 10000 × g for 5 min to remove non-bound IgA. The bacteria pellet was resuspended in 200 μL buffer containing SYBR Green I (Invitrogen catalog number S7563) at a 1:10000 dilution for 15 min at 4°C, followed by bacteria staining with PE-conjugated anti-mouse IgA prior to flow cytometric analysis.

### Flow cytometry for immune cells

Small intestine and colon tissues were digested with Collagenase IV (Yeasen Biotech Co., Ltd., Shanghai, China) at 37°C for 2 h, then filtered through a 70-μm cell strainer (Corning Incorporated, Corning, NY, USA) to obtain the single cells. MLNs were gently ground and then suspended in PBS, followed with filtering through a 70-μm cell strainer to obtain single cells. The single-cell suspensions were then incubated in the dark with anti-mouse antibodies to CD45.2 and CD45R. Intracellular staining was performed using a Foxp3 Staining Buffer Kit (eBioscience catalog number 00-5523-00) for IgA marker. Analysis was performed immediately by using an FACSCalibur™ flow cytometer (Becton-Dickinson, Fullerton, CA, USA).

### Quantitative real-time polymerase chain reaction for gene expression analysis

Total RNA was extracted from the small intestine tissues using TRIzol™ Reagent (Ambion catalog number 15596026), converted to cDNA by *EasyScript^®^* All-in-One First-Strand cDNA Synthesis SuperMix for qPCR (catalog number AE341-03, TransGen Biotech, Beijing, China), and then used for RT-PCR. Quantitative RT-PCR was performed with TB Green Premix Ex Taq II (catalog number RR820A, TaKaRa Bio. Inc., Shiga, Japan). Relative gene expression level of targets was calculated by 2^−ΔΔCt^ method (20), and β-actin was used as the reference gene. The specific primers used are indicated in **Table S2**.

### Transcriptome sequencing analysis

The total RNA extraction, whole transcriptome libraries preparation, and RNA sequencing were performed by the Majorbio Bio-pharm Technology Co., Ltd. (Shanghai, China). Briefly, total RNA was extracted from the tissue using TRIzol™ Reagent according the manufacturer’s instructions and genomic DNA was removed using DNase I (TaKara). RNA-seq transcriptome library was prepared following TruSeq™ RNA sample prep Kit (Illumina, San Diego, CA, USA), and was subsequently sequenced with the Illumina HiSeq X™ Ten. The raw paired end reads were trimmed and quality controlled by SeqPrep and Sickle with default parameters. Then the high-quality clean reads were aligned to the reference genomes, Mus musculus (GRCm38.p6), using HISAT2. RSEM 1.3.3 was used to quantitatively analyze the expression levels of genes based on transcripts per million (TPM), and edgeR 3.14.0 was used to identify the differentially expressed genes (DEGs) between the control group and the anthocyanin group. If the *p*-value was less than 0.05 and the fold change greater than or equal to 2, it was considered to be a significantly different expression level.

### ROS detection

Single-cell suspensions of small intestine and colon tissues were obtained as described above after two-week anthocyanin dietary intervention. The harvested cells were washed twice with PBS, and then collected and suspended in the diluted DCFH-DA probe (diluted with serum-free cell culture medium at 1:1000 to a final concentration of 10 μM; catalog number CA1410, Solarbio Science and Technology Co., Ltd., Beijing, China), and incubated in a cell incubator at 37°C for 20 min. Then the cells were washed three times with serum-free cell culture medium before flow cytometry analysis.

In addition, *in situ* ROS imaging by Pannoramic MIDI (3DHISTECH Ltd., Budapest, Hungary) was performed using dihydroethidium (DHE) as probe for intracellular ROS as VACCARO A et al. previously described (21) and DAPI (blue) for visualization of cell nuclei.

### Fecal redox potential detection

0.1 g freshly voided feces were suspended in 1 mL of ice-cooled ultrapure water, and homogenized by beat beating with 1 mm zirconium beads, then the redox potential of the fecal homogenate samples was measured using a simple redox probe (Mettler-Toledo International Inc., Greifensee, Switzerland).

### Bioinformatics analysis

The alpha diversity was calculated respectively for each group by package vegan and plotted with package ggplot2 in R 3.6.1. For beta-diversity (sample-to-sample dissimilarity), the principal component analysis (PCA) on genus level was performed using R package ade4 and illustrated with R package made4. PICRUSt (phylogenetic investigation of communities by reconstruction of unobserved states) was used to evaluate the functional potential of microbial communities (22). Organism-level microbiome phenotypes were predicted with BugBase (23). WGCNA (24)was used to identify key modules and hub bacterial taxa associated with phenotypes, such as the secretion of sIgA and antimicrobial peptides in small intestine, fecal free IgA.

### Statistical analysis

Statistical analyses were carried out using GraphPad Prism 8.0 (GraphPad Software Inc., USA). All continuous variables were presented as means ± SEM and compared between groups using Student’s *t*-test unless otherwise indicated. *p* < 0.05 was considered to be statistically significant. * denotes *p* < 0.05, ** denotes *p* < 0.01, and *** denotes *p* < 0.001, p > 0.05 was considered to be not Statistically Significant (ns).

## Results

### Impacts of anthocyanins on small intestine transcriptome

**Figure 1:**
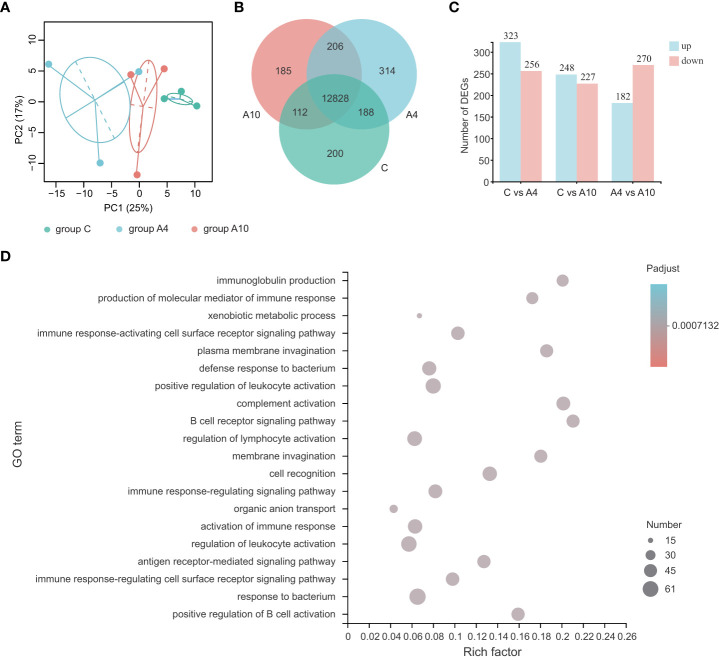
Anthocyanins induce major changes in the transcriptome of the small intestine of mice. **(A)** The results from the PCA analysis of the small intestine RNA-sequencing data (n = 3, 95% confidence interval). **(B)** The Venn analysis of expressed genes (transcripts Per Million reads ≥ 1) between groups. **(C)** The numbers of DEGs between groups. **(D)** The top 20 ranked GO terms according to the rich factor of DEGs between group C and group A4 (n = 3), adjusted *p* value < 0.05 (corrected for multiple hypothesis testing with the Benjamini–Hochberg method). The vertical axis indicates GO terms, the horizontal axis represents the rich factor, and the size of dots indicates the number of DEGs accumulated in the GO terms. group C, the control group; group A4, the 4-day anthocyanin treatment group.

Firstly, we performed small intestine transcriptome analysis to assess the influence of anthocyanins on the gut of mice. Here, PCA analysis (**Figure 1A**) reveals the clustering of samples from the control and anthocyanin-treated groups by treatment types. The Venn diagram (**Figure 1B**) was applied to show specifically expressed and invariant genes between the control group (group C), the 4-day anthocyanin treatment group (group A4) and the 10-day anthocyanin treatment group (group A10). Further identifying DEGs, group C and group A4 had 549 DEGs, group C and group A10 had 475 DEGs, while group A4 and group A10 had 452 DEGs (**Figure 1C**).

Next, we performed GO (Gene Ontology) enrichment analysis and KEGG (Kyoto Encyclopedia of Genes and Genomes) enrichment analysis for the DEGs. The GO enrichment analysis of DEGs for group C and group A4 suggested enriched GO terms associated with the immune response (**Figure 1D**). For example, the GO terms affiliated with both the defense and responses to bacterium were significantly influenced by anthocyanin supplement (**Figure 1D**). Especially, the GO term immunoglobulin production was significantly influenced by 4-day anthocyanin supplement, which also affected the KEGG pathway intestinal immune network for IgA production (**Figure S1**). Similarly, 10-day anthocyanin supplement significantly influenced the GO terms associated with the immune response (**Figure S2**).

### Effects of anthocyanins on the secretion of lysozyme and sIgA

**Figure 2:**
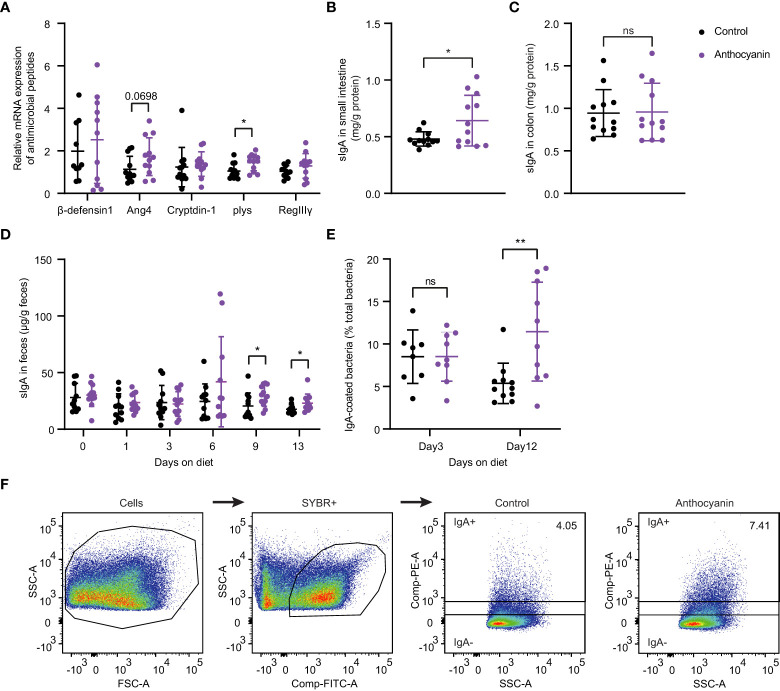
Anthocyanins influence the secretion of antimicrobial peptides and sIgA, the fecal free-IgA concentration and the IgA-coated bacteria. **(A)** The relative mRNA expression of antimicrobial peptides was expressed as fold changes against β-actin. sIgA concentration in small intestine **(B)** and in colon **(C)**. For above analysis, the samples were collected after two-week anthocyanin treatment (on the 14th day). **(D)** Changes of fecal free-IgA concentration with the time of anthocyanin dietary intervention (Mann-Whitney test). **(E)** The proportion of IgA-coated bacteria on the 3rd and 12th day of anthocyanin dietary intervention. **(F)** Gating strategy for IgA-coated bacteria. Fecal bacteria from male C57BL/6 mice were stained with PE-conjugated anti-mouse IgA and gated based on FSC/SSC characteristics and fluorescence of SYBR^+^ (FITC). IgA^+^ and IgA^−^ populations were gated from SYBR^+^ bacteria. Data are means ± SEM. * denotes *p* < 0.05, ** denotes *p* < 0.01 (Student’s *t*-test). p > 0.05 was considered to be not Statistically Significant (ns).

To validate the findings from the RNA-seq analysis, we investigated gene expression for several antimicrobial peptides in small intestine using quantitative RT-PCR. As shown in **Figure 2A**, no difference could be identified among the expressions of β-defensin-1, RegIII-γ (regenerating islet-derived protein 3-gamma), and cryptdin-1 between the control group and the anthocyanin group. However, the expression of Ang4 (angiogenin 4), a Paneth cell-derived antimicrobial peptide (25), was slightly enhanced by anthocyanin intake. Meanwhile, anthocyanins significantly increased the gene expression for lysozyme (plys) secreted by Paneth cells. The ELISA assay showed that the level of sIgA in small intestine was significantly increased after two-week anthocyanin dietary intervention (**Figure 2B**), while there was no significant effect on the level of sIgA in colon (**Figure 2C**). **Figure 2D** showed the change of the fecal free-IgA concentration over time. On the 0th,1st, and 3rd day of the anthocyanin dietary intervention, there was no difference between the control group and the anthocyanin group. From the 6th day, the anthocyanin group showed a higher fecal free-IgA concentration, while there was a significant difference on the 9th day and the 13th day. For the serum samples, neither IgA nor IgG showed statistical difference in concentration between the control group and the anthocyanin group, while IgM concentration was significantly increased in the anthocyanin group (**Figure S3**). These findings indicate that anthocyanins promote the production of sIgA in the small intestinal.

The IgA-coated bacteria from fecal materials were analyzed by flow cytometry. Although we observed no differences in the median fluorescence intensity (MFI, flow cytometry) of IgA-coated bacteria between the anthocyanin group and the control group on either day 3 or day 12 (**Figure S4**), the proportion of IgA-coated bacteria in the anthocyanin group was significantly increased on day 12 (**Figure 2E**). The gating strategy for IgA-coated bacteria was shown in **Figure 2F**.

### Anthocyanins increase IgA-producing plasma cells possibly through upregulating expression of TGF-β1

**Figure 3:**
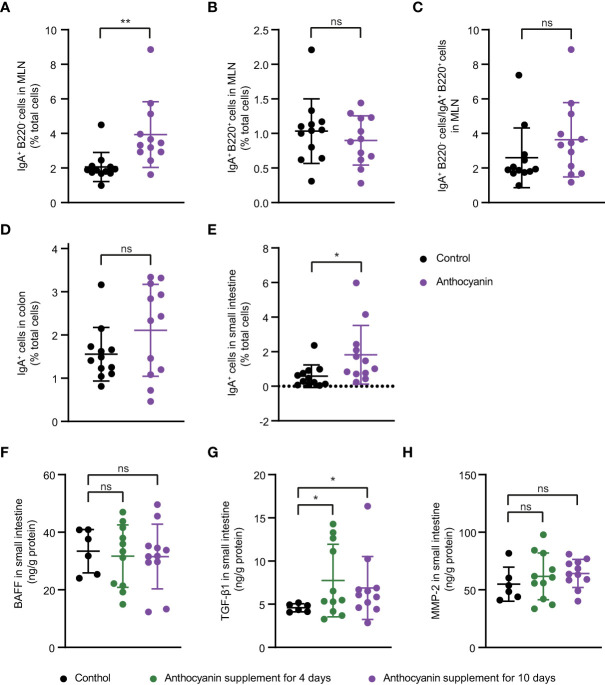
Possible mechanisms underlying the increased secretion of IgA induced by anthocyanins. IgA-producing plasma cells (IgA^+^ B220^−^) **(A)**, IgA-producing B cells (IgA^+^ B220^+^) **(B)** and IgA^+^ B220^−^ cells to IgA^+^ B220^+^ cells ratio **(C)** in the MLNs. Percentage of IgA^+^ cells in small intestine **(D)** and in colon **(E)**. For **(A–E)**, samples were collected after oral administration of bilberry anthocyanins for two weeks. Concentration of BAFF **(F)**, TGF-β1 **(G)** and MMP-2 **(H)** in small intestine were analyzed after 4-day or 10-day anthocyanin intake. Data are means ± SEM. * denotes *p* < 0.05, ** denotes *p* < 0.01 (Student’s *t*-test). p > 0.05 was considered to be not Statistically Significant (ns).

The intestinal IgA^+^ immune cells were also determined with flow cytometer. Anthocyanins significantly increased the percentage of IgA-producing plasma cells (IgA^+^ B220^−^) in the MLNs (**Figure 3A**), while did not affect the percentage of IgA-producing B cells (IgA^+^ B220^+^) (**Figure 3B**). Compared with the control group, the anthocyanin group appeared a higher IgA^+^ B220^−^ cells/IgA^+^ B220^+^ cells ratio (**Figure 3C**), although there is no significant difference between them (*p* = 0.0996, Kolmogorov-Smirnov test). The gating strategy for IgA-producing plasma cells and IgA-producing B cells was shown in **Figure S5**. The proportion of IgA^+^ cells was significantly increased (**Figure 3D**) in the small intestine, whereas a minor increase of IgA^+^ cells appeared in the colon (**Figure 3E**).

Both class switching of B cells and differentiation of plasma cells are conditioned by certain cytokines, especially TGF-β1 and BAFF (26). Here, we analyzed the expression of TGF-β1 and BAFF at protein levels in small intestine. BAFF showed no difference between the control group and the anthocyanin group (**Figure 3F**), while anthocyanins significantly enhanced the expression of TGF-β1 (**Figure 3G**). MMP-2, a type of matrix metalloproteinases (MMPs) that mediate conversion of TGF-β1 from inactive to active form (26), was also increased in the anthocyanin group, although there was no statistical difference (**Figure 3H**). Overall, the increased IgA-producing plasma cells may be due to the enhanced expression of TGF-β1.

### Anthocyanin changes the composition of gut microbiota at different taxonomic levels

**Figure 4:**
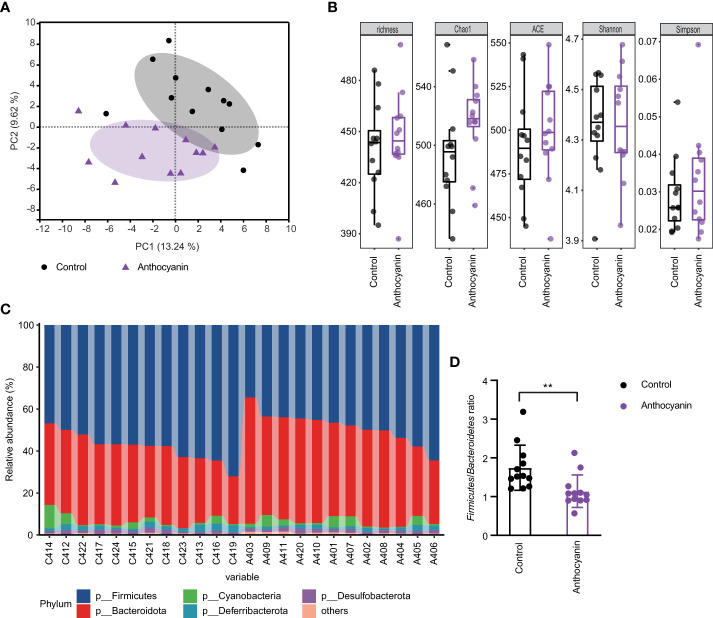
Oral administration of bilberry anthocyanins changes the mouse gut microbiota structure. **(A)** Beta-diversity analysis of microbial communities by using PCA at genus level. **(B)** For alpha diversity at OTU level, richness, Chao1, ACE, Shannon, and Simpson indices were calculated. **(C)** The relative abundance of gut microbiota at the phylum level in each sample. C denotes the control group and A denotes the anthocyanin group. **(D)** The *Firmicutes* to *Bacteroidetes* ratio. Data are means ± SEM. ** denotes *p* < 0.01 (Student’s *t*-test).

The samples for mice cecal contents were collected for 16S rRNA sequencing after 2-week treatment. We compared the community structure of each group at different taxonomic levels. As indicated in **Figure 4A**, PCA analysis showed obvious changes (R^2^ = 0.1466, *p* = 0.013, Adonis variance analysis based on Euclidean distance at genus level) in the composition of gut microbiota after oral supplement of anthocyanins. For the alpha diversity at OTU level, the richness, Chao1, and ACE indices were increased in the anthocyanin group compared with the control group, although there was no statistical difference (**Figure 4B**). At the phylum level, *Firmicutes* and *Bacteroidetes* were the most dominant commensals in the gut microbiota of the two groups (**Figure 4C**). Anthocyanin supplement dramatically increased the relative abundance of *Bacteroidetes* (*p* = 0.0038, Student’s *t*-test) while decreased the *Firmicutes* (*p* = 0.0019, Student’s *t*-test). The *Firmicutes* to *Bacteroidetes* (F/B) ratio was significantly decreased in the anthocyanin group compared to the control group (**Figure 4D**). Several studies showed an association between the F/B ratio and obesity (27). As expected, anthocyanins supplement led to a significant reduction of weight gain compared with the control group (**Figure S6**). Moreover, the abundance of the unknown family of the order *Clostridia_vadinBB60_group* was also significantly reduced (*p* = 0.01, Wilcoxon rank-sum test), while an unclassified genus belonging to the *VadinBB60* family is positively correlated with feed efficiency (28). This may also partially explain the decreased weight gain, suggesting that anthocyanins could influence energy metabolism of gut microbiota.

### Fecal free IgA levels are associated with certain bacterial species

**Figure 5:**
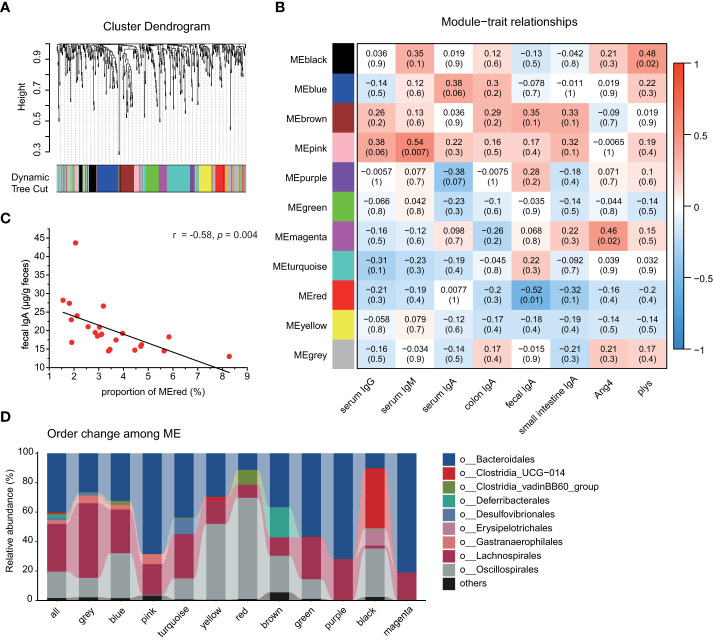
Weighted gene co−expression network analysis identifies key modules associated with phenotype. **(A)** Cluster dendrogram. Each color represents one specific co−occurrence module, and branches above represent OTUs. **(B)** Heatmap of the correlation between interested phenotypes and module eigenOTUs. Each column corresponds to a phenotype, and each row corresponds to a module. Each cell contains the correlation coefficients which correspond to the cell color, blue represents negative correlation and red represents positive correlation. The *p*−values are stated in the brackets. **(C)** Correlation analysis of fecal IgA and the proportion of the red module, Pearson correlation coefficient; **(D)** Order level taxonomic composition of the co−occurrence modules.

We also investigated the association between the gut microbiota and the secretion of immunoglobulins and antimicrobial peptides by the WGCNA analysis. A soft threshold was set to 7 in order to meet the degree of independence of 0.85 with the minimum power value (**Figure S7**). Then, we constructed modules and performed dynamic branch cutting with a merging threshold of 0.25. 11 modules were obtained (**Figure 5A**). The module−trait associations were analyzed by correlating module with experimental traits to identify significant associations (**Figure 5B**). The magenta module and the black module were positively correlated with Ang4 (r = 0.46; *p* = 0.02) and plys (r = 0.48; *p* = 0.02), respectively. The pink module was positively correlated with serum IgM concentration (r = -0.54; *p* = 0.007). As shown in **Figure 5C**, the red module was negatively correlated with the fecal free-IgA (r = -0.58; *p* = 0.004; Pearson correlation coefficient). Compared with the composition of all bacteria (**Figure 5D**), *Clostridia_vadinBB60_group* and *Oscillospirales* were significantly increased in the red module (*p* < 0.0001, Student’s *t*-test). Whereas the abundance of these two orders decreased in the anthocyanin group, *Clostridia_vadinBB60_group* showed significant difference (**Figure S8**). In addition, Pearson correlation coefficient (r = -0.54; *p* = 0.03) further confirmed the negative correlation between the abundance of *Clostridia_vadinBB60_group* and the fecal IgA.

### Anthocyanins increase anaerobic bacteria and downregulate ROS levels in the gut

**Figure 6:**
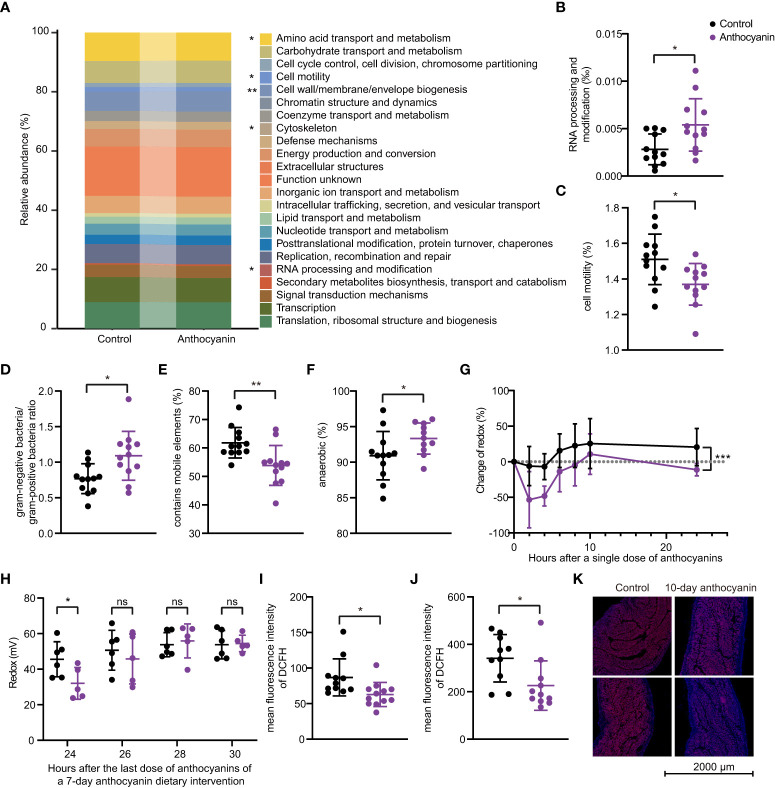
The PICRUSt and BugBase analyses based on 16S rRNA gene sequences. **(A)** PICRUSt prediction of functional profiling of the microbial communities. Significant differences exist between the control group and the anthocyanin group in the RNA processing and modification module **(B)** and the Cell motility module **(C)**. The BugBase analysis showed significant differences in the proportion of anaerobic bacteria **(D)**, the gram-negative bacteria to gram-positive bacteria ratio **(E)** and the relative abundance of taxa containing mobile elements **(F)**. **(G)** Change of fecal redox potential after a single dose of anthocyanins compared to the initial value. Data are means ± SEM, n = 6 per group, repeated measures ANOVA (time × change of redox) with Sidak′s multiple. Change of redox = (R_test_ – R_initial_)/R_initial_ *100, R_initial_ denotes the redox potential before the anthocyanin intake, R_test_ denotes redox potential measured at different time points after a single dose of anthocyanins. **(H)** Changes in fecal redox potential with time after the last dose during a 7-day anthocyanin dietary intervention. Intracellular ROS levels in small intestine **(I)** and in colon **(J)** were determined through DCFH-DA ROS probe by flow cytometry, and samples were collected after two-week anthocyanin treatment. **(K)** Representative *in situ* ROS imaging of colon after 10-day anthocyanin treatment. Data are means ± SEM. * denotes *p* < 0.05, ** denotes *p* < 0.01 (Student’s *t*-test). p > 0.05 was considered to be not Statistically Significant (ns).

To investigate how anthocyanins trigger improvement in the gut microbiota, we performed PICRUSt and BugBase analysis based on 16S rRNA sequencing data. **Figure 6A** shows the results from the PICRUSt analysis. Some COGs (Cluster of Orthologous Groups of proteins) showed significant changes, especially the RNA processing and modification module (**Figure 6B**), the relative abundance of which was significantly enhanced in the anthocyanin group (*p* = 0.011). The relative abundance of Cell wall/membrane/envelope biogenesis was significantly enhanced in the anthocyanin group (*p* = 0.0011). However, the cell motility module, which plays an important role in promoting bacterial diversity (29), is significantly reduced in the anthocyanin group (**Figure 6C**). The BugBase analysis indicated that anthocyanins significantly increased the abundance of gram-negative bacteria (*p* = 0.012, Student’s *t*-test) while decreased gram-positive bacteria (*p* = 0.012, Student’s *t*-test) abundance. In other words, anthocyanins resulted in a significant enhanced ratio of gram-negative bacteria to gram-positive bacteria (**Figure 6D**). Meanwhile, we noticed that the anthocyanin group appeared a significant reduction in the relative abundance of taxa containing mobile elements (**Figure 6E**). In addition, as shown in **Figure 6F**, the proportion of anaerobic bacteria significantly increased (*p* = 0.0492, Kolmogorov-Smirnov test). A significant reduction in mouse fecal redox potential following anthocyanin supplement (**Figures 6G** **, H**) suggested a possible improvement in the intestinal anaerobic environment by anthocyanins and thus influence the gut microbiota. Moreover, both flow cytometry analysis and *in situ* ROS imaging showed that anthocyanins could significantly reduce the ROS levels in the small intestine and in the colon (**Figures 6I**–**K**).

It was hypothesized that the increased cell wall/membrane/envelope biogenesis and the abundance of gram-negative bacteria may contribute to LPS production (30), which ultimately affects the host immune response. Here we detected the LPS levels in feces and serum, and no influence was identified on the LPS levels (**Figure S9**). In addition, anthocyanins did not influence the expression of mucin 2 (Muc-2) and tight junction proteins including occludin, claudin-1, and zonula occludens-1 (ZO-1) in small intestine (**Figure S10**), or the levels of inflammatory cytokines IL-6 and TNF-α (**Figure S11**) and the fecal concentrations of acetate, propionate and *n*-butyrate (**Figure S12**).

## Discussion

*In vitro* fermentation studies showed that both anthocyanins mixture (8, 31) and anthocyanin monomers (8, 32, 33) could modulate the gut microbiota. Studies on animals (9, 34) or in human subjects (35) further confirm the prebiotic potential of anthocyanins. Here, we found that anthocyanins are favorable for maintaining the anaerobic intestinal environment of mice, promoting the secretion of secretory immunoglobulin A (sIgA) and antimicrobial peptides, and directly or indirectly altering bacterial physiology, including the cell motility and mobile genetic elements. These attractive properties may account for the beneficial gut microbiota modulating activity of anthocyanins.

In the present study, we observed a significant reduction in mouse fecal redox potential following anthocyanin supplement. Meanwhile, the Bugbase analysis show that the proportion of anaerobic bacteria significantly increased in the anthocyanin group, which was consistent with our previous results (12). These findings confirmed that anthocyanins might influence gut microbiota through maintaining the anaerobic intestinal environment. In addition, anthocyanins significantly downregulated the cell motility and mobile genetic elements. Because bacterial motility differences and their trade-offs with growth are adequate to promote diversity (29), while mobile genetic elements contribute to bacterial adaptation and evolution (36), the effect of anthocyanins on the bacterial motility and mobile genetic elements may also be one of factors responsible for the gut microbiota modulating activity. Moreover, we observed that anthocyanins promoted the secretion of sIgA and antimicrobial peptides, both of them help defend against the infection of pathogens and potentially harmful commensal bacteria (37), and are crucial for establishing a balanced microbiota (38, 39). There might be another way that anthocyanins may display healthy attributes. It was reported that sIgA downregulated genes involved in bacterial motility and reduced motility *in vitro* in a plate motility assay (40), suggesting that anthocyanins might indirectly influence the composition and function of the gut microbiota by promoting sIgA secretion.

It has been reported that anthocyanins could increase salivary mucosal sIgA level of trial participants (41), IgA level in cecal content of male Fischer-344 rats (17), and the level of sIgA in the bronchoalveolar lavage fluids and feces of female BALB/c mice (18). Here, we found that anthocyanins can significantly promote the secretion of sIgA in the small intestine, which might be possibly through the induction of the maturation of IgA-producing plasma cells. sIgA secreted to the lumen, as an important component of the mucosal barrier, works by coating and agglutinating its targets, which avoids potentially harmful stimulation of the intestinal mucosal immune system (38). In addition, IgA is effective in killing tumor cells by recruiting neutrophils, and has strong activity against several pathogens, including rotavirus, poliovirus, influenza virus, and SARS-CoV-2 (42). In cases where IgM and IgG levels are normal and there is no other cause of hypogammaglobulinemia, the isolated IgA deficiency is defined as selective immunoglobulin a deficiency (SIgAD), which is the most common primary immunodeficiency and have a significant association with mucosal infection, increased risks of atopic disease, and a higher prevalence of autoimmune disease (43). Considering the multifaceted functions IgA, anthocyanins may be useful for preventing gastrointestinal infections and immunotherapy.

To date, the biochemical basis for the gut microbiota modulating activity of anthocyanins remains inadequately understood. The gut microbial-dependent metabolism of anthocyanins has been well documented and the consumption of anthocyanins has been considered as one important mechanism responsible for the regulation of the intestinal bacterial growth and the colonization of the gut microbiota (5). In addition to this, it is reported that blackcurrant anthocyanins can obviously reduce the gastrointestinal luminal oxygen levels and thus promoting growth of oxygen-sensitive bacterial populations (44). In the present study, we found that anthocyanins could not only reduce the fecal redox potential and increase the proportion of anaerobic bacteria, but also significantly downregulate the ROS levels in the gut. Considering that ROS has a profound influence on the class-switch recombination and plasma cell differentiation (45), we expect that the antioxidant effects of anthocyanins may play a vital role in the regulation of sIgA secretion and the consequent modulation of the gut microbiota. All these hint that the antioxidant activity of anthocyanins might be an important biochemical basis for their prebiotic property.

## Conclusion

In the present study, we found that oral administration of anthocyanins can significantly promote the secretion of sIgA and antimicrobial peptides in the small intestine of mice. The increase of the proportion of IgA-producing plasma cells in the MLNs is possibly through inducing the expression of TGF-β1. Further study showed that anthocyanins could obviously reduce the fecal redox potential while increasing the proportion of anaerobic bacteria and downregulate ROS levels in the gut. Moreover, anthocyanins can markedly change the relative abundance of specific bacteria (such as the order *Clostridia_vadinBB60_group*) and the function of the gut microbiota (such as the cell motility and mobile genetic elements of commensal bacteria). All these findings suggest that, as natural antioxidants, anthocyanins have a promising potential in maintaining the intestinal homeostasis. These phytochemicals might find unpresented applications in the treatment of human diseases related to bacterial dysbiosis.

## Data availability statement

The datasets presented in this study can be found in online repositories. The names of the repository/repositories and accession number(s) can be found below: https://www.ncbi.nlm.nih.gov/, PRJNA780796, https://www.ncbi.nlm.nih.gov/, PRJNA780807.

## Ethics statement

This study was conducted according to the guidelines of the Institutional Animal Care and Use Committees of Tsinghua University for animal welfare (approval ID No. 20-LZ1#).

## Author contributions

Funding acquisition, GJ and ZL. Investigation, experiments design, data analysis and figure preparation, LW and XL. Writing—original draft, XL. Writing—review and editing, LW, GJ and ZL. XL performed the most experiments. HZ and ZY contributed to some experiments. All the authors read and approved the final manuscript. All authors contributed to the article and approved the submitted version.

## Funding

This work was funded by Chunfeng Foundation of Tsinghua University, Xinjiang Tianhui Information Technology Co. Ltd., Xinjiang Tianjianhemu Biotech Co. Ltd. under project contract No. 2021Z99CFZ011, No. 20192000168 and No. 20172000941. The funders was not involved in the study design, collection, analysis, interpretation of data, the writing of this article or the decision to submit it for publication.

## Acknowledgments

The authors would like to acknowledge their sincere thanks to Prof. Jianzhong Wu from Department of Chemical and Environmental Engineering, University of California, Riverside, USA, for valuable comments and assistance in preparing this manuscript.

## Conflict of interest

The authors declare that the research was conducted in the absence of any commercial or financial relationships that could be construed as a potential conflict of interest.

## Publisher’s note

All claims expressed in this article are solely those of the authors and do not necessarily represent those of their affiliated organizations, or those of the publisher, the editors and the reviewers. Any product that may be evaluated in this article, or claim that may be made by its manufacturer, is not guaranteed or endorsed by the publisher.
