# Supplementary material for: Promoting intestinal IgA production in mice by oral administration with anthocyanins
Source: Front Immunol. 2022 Jul 27;13:826597. doi: 10.3389/fimmu.2022.826597 (PMC9364608; doi:10.3389/fimmu.2022.826597)
Supplement: Supplementary file 1 [file DataSheet_1.docx]

Supplementary Material

# Supplementary Figures and Tables

## Supplementary Figures


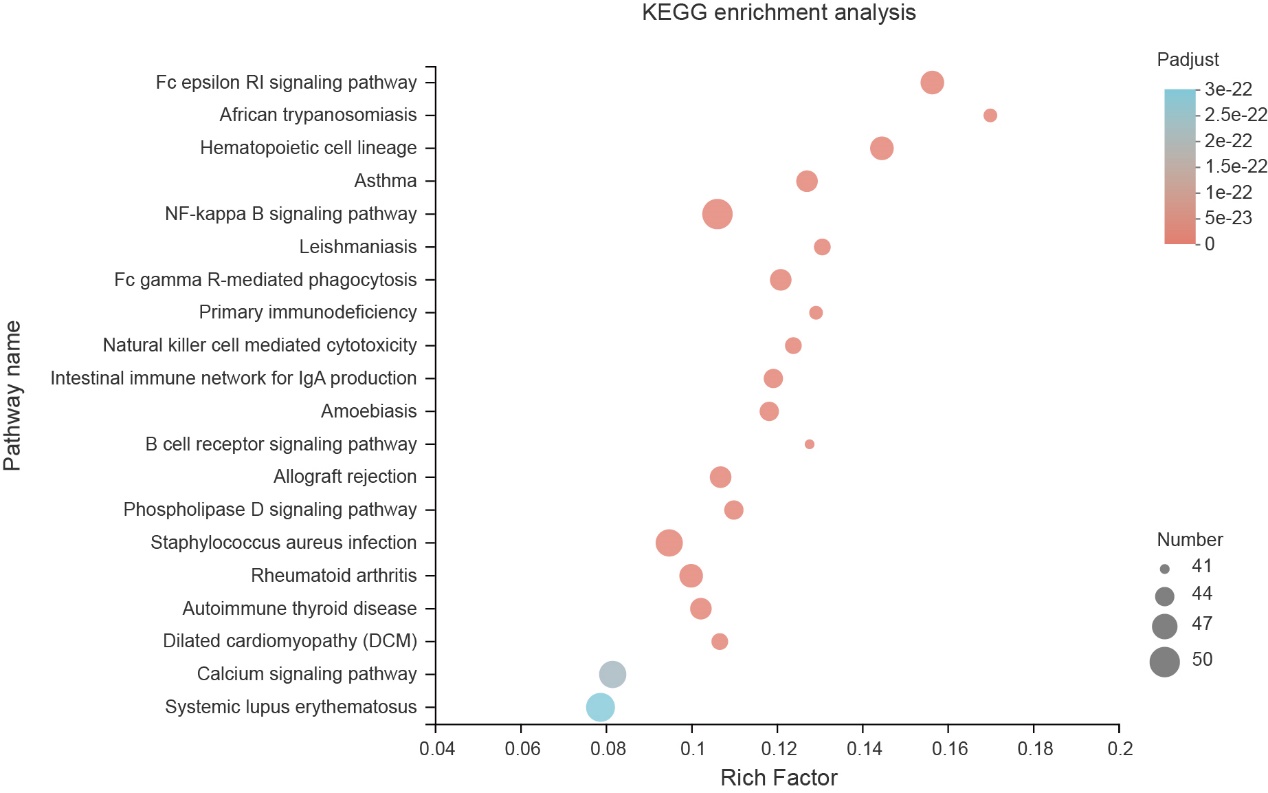


**Supplementary Figure S1.** **The KEGG enrichment analysis of differentially expressed genes (DEGs) between the group C and the group A4.** The top 20 ranked KEGG pathways according to the rich factor of DEGs between group C and group A4 (n = 3), adjusted *p* value < 0.05 (corrected for multiple hypothesis testing with the Benjamini-Yekutieli method). The vertical axis indicates KEGG pathways, the horizontal axis represents the rich factor, and the size of dots indicates the number of DEGs accumulated in the KEGG pathway. group C, the control group; group A4, the 4-day anthocyanin treatment group.


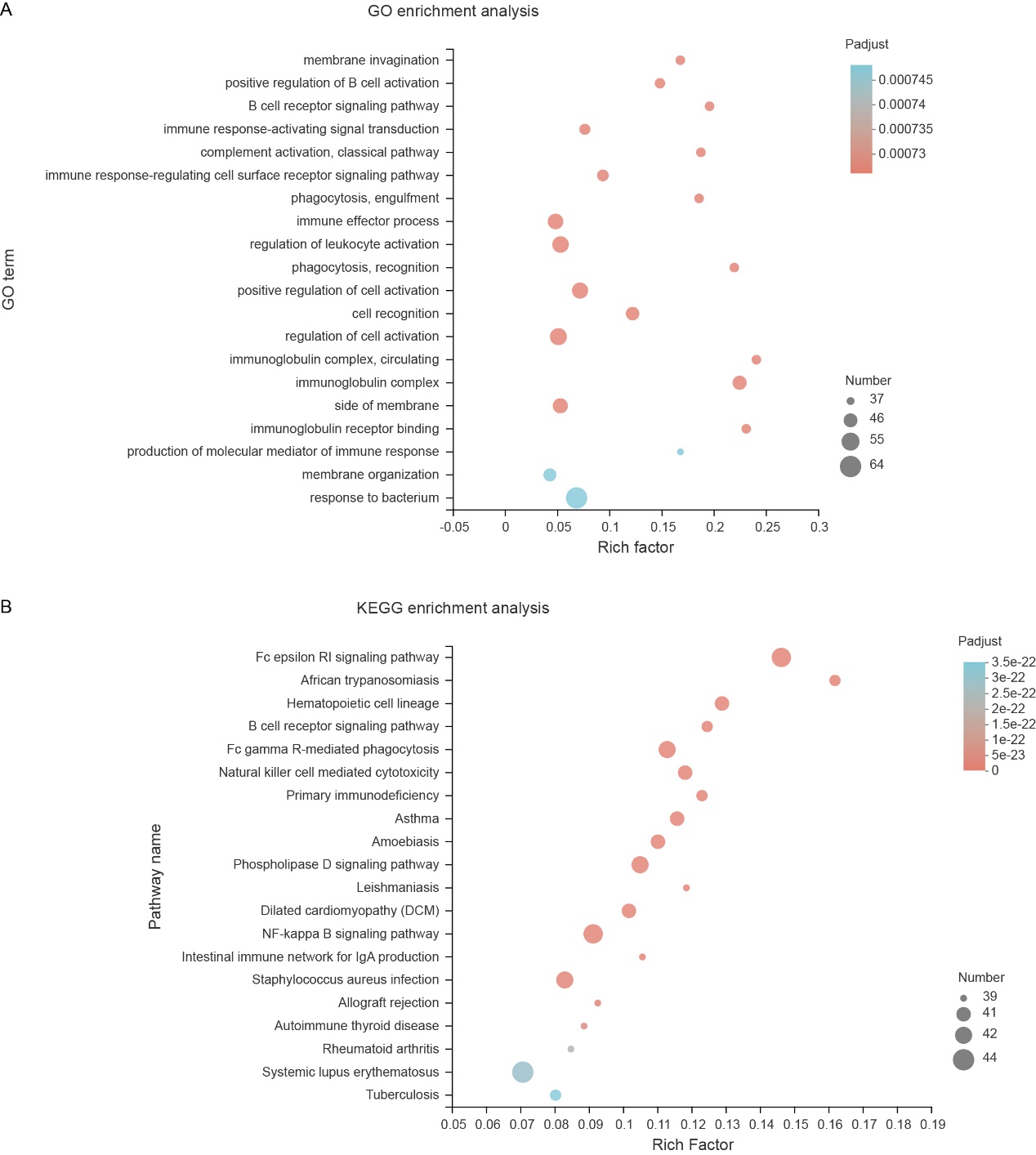


**Supplementary Figure S2.** **Functional enrichment analysis of DEGs between the group C and the group A10.** (A) The top 20 ranked GO terms according to the rich factor of DEGs between group C and group A10 (n = 3), adjusted *p* value < 0.05 (corrected for multiple hypothesis testing with the Benjamini–Hochberg method). The vertical axis indicates GO terms, the horizontal axis represents the rich factor, and the size of dots indicates the number of DEGs accumulated in the GO term. group C, the control group; group A10, the 10-day anthocyanin treatment group. (B) The top 20 ranked KEGG pathways according to the rich factor of DEGs between group C and group A10 (n = 3), adjusted *p* value < 0.05 (corrected for multiple hypothesis testing with the Benjamini-Yekutieli method). The vertical axis indicates KEGG pathways, the horizontal axis represents the rich factor, and the size of dots indicates the number of DEGs accumulated in the KEGG pathway.


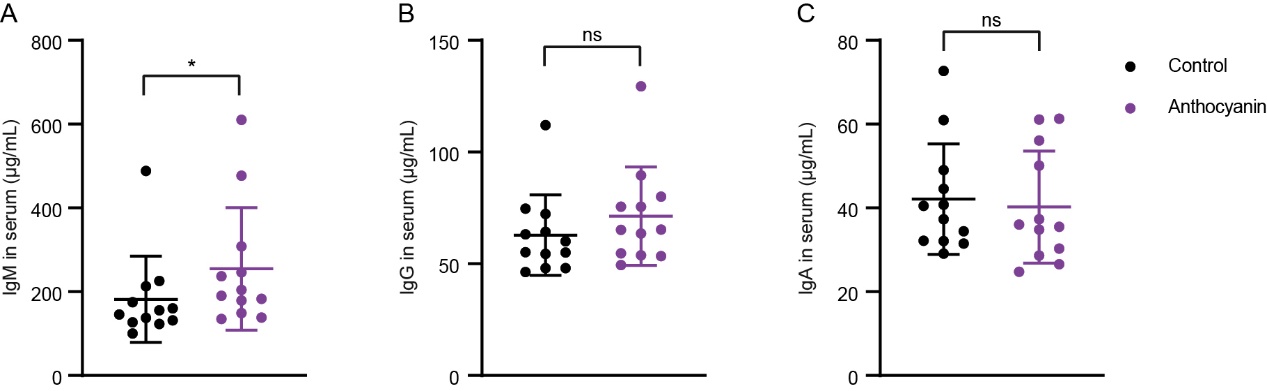


**Supplementary Figure S3.** **Effects of anthocyanins on the level of immunoglobulins in serum.** IgM (A), IgG (B) and IgA (C) levels in serum after two-week anthocyanin dietary intervention. Data are means ± SEM. * denotes *p* < 0.05 (Mann-Whitney test).


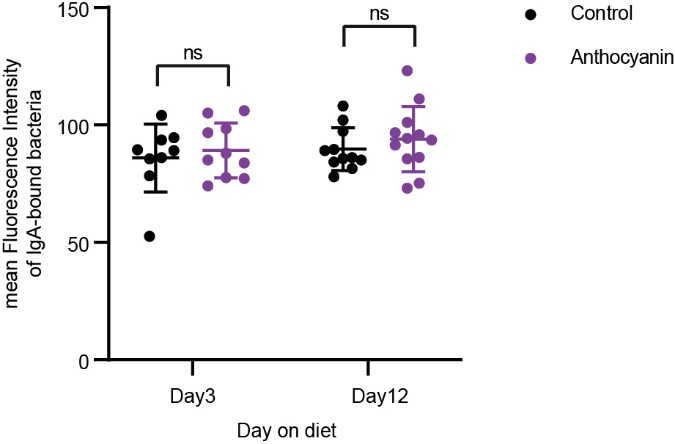


**Supplementary Figure S4.** **The median fluorescence intensity of IgA-coated bacteria on day 3 and day 12 of anthocyanin dietary intervention.** Data are means ± SEM. Student’s *t*-test.


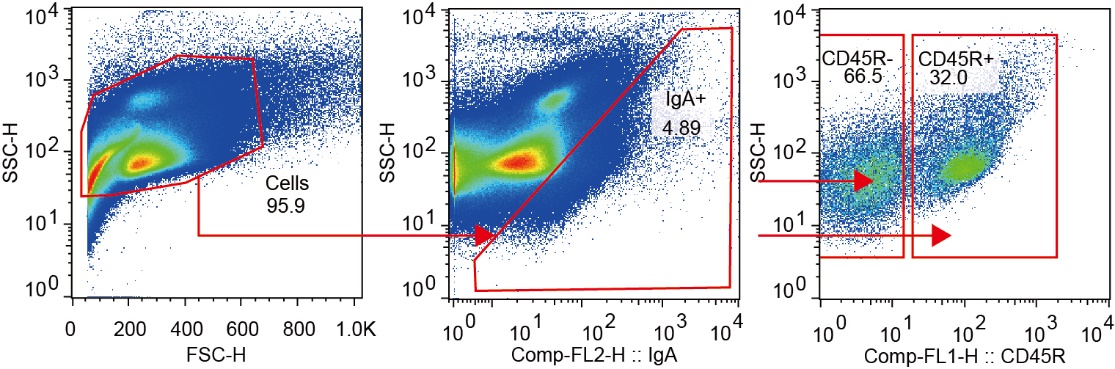


**Supplementary Figure S5. The gating strategy for IgA-producing plasma cells and IgA-producing B cells**.


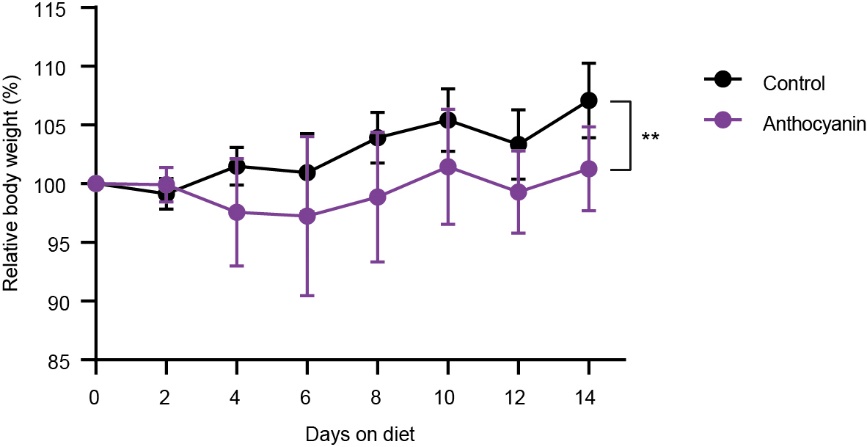


**Supplementary Figure S6.** **Effects of bilberry anthocyanins on body weight gain.** Data are means ± SEM. * denotes *p* < 0.05, ** denotes *p* < 0.01, Repeated measures ANOVA (time × weight) with Sidak′s multiple.


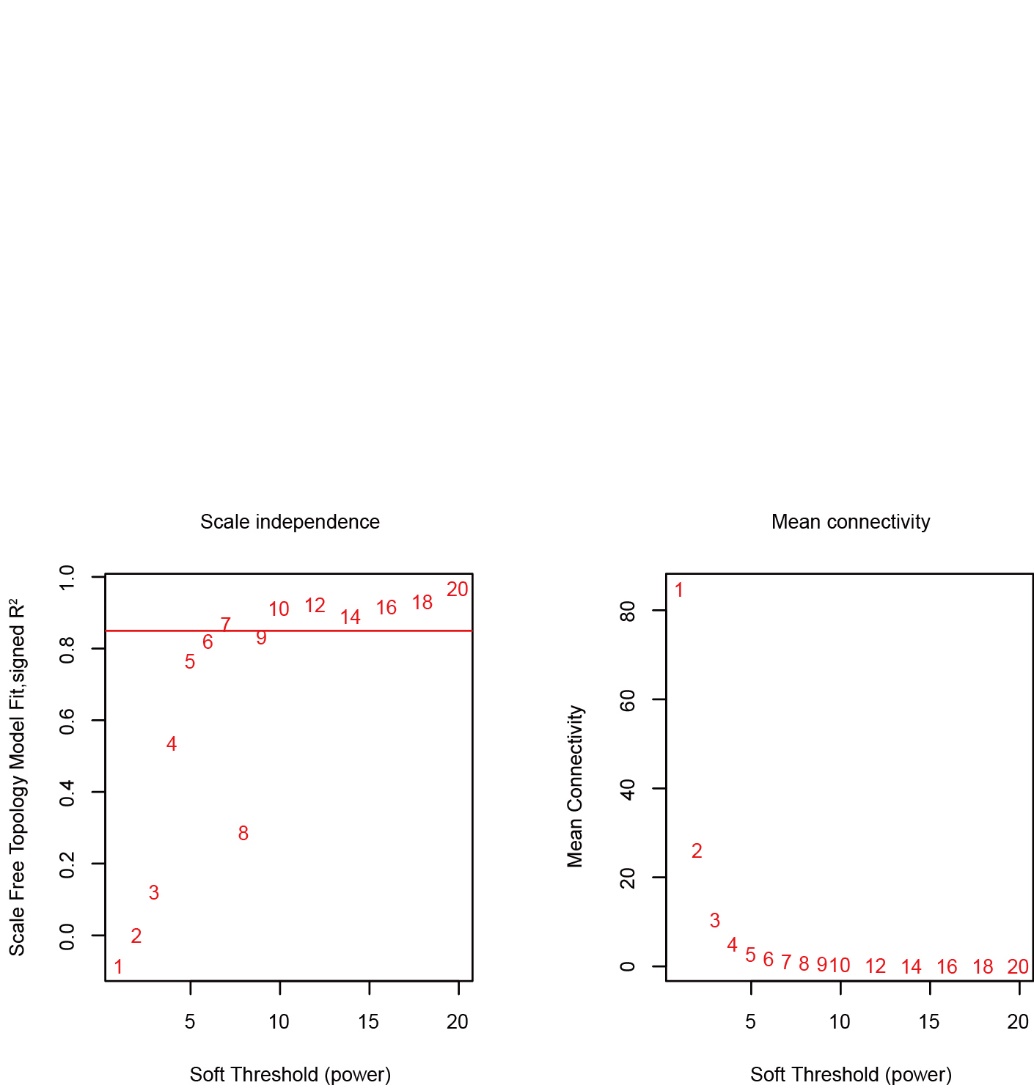


**Supplementary Figure S7.** **Soft threshold selection process for the construction of weighted co‑expression network.**


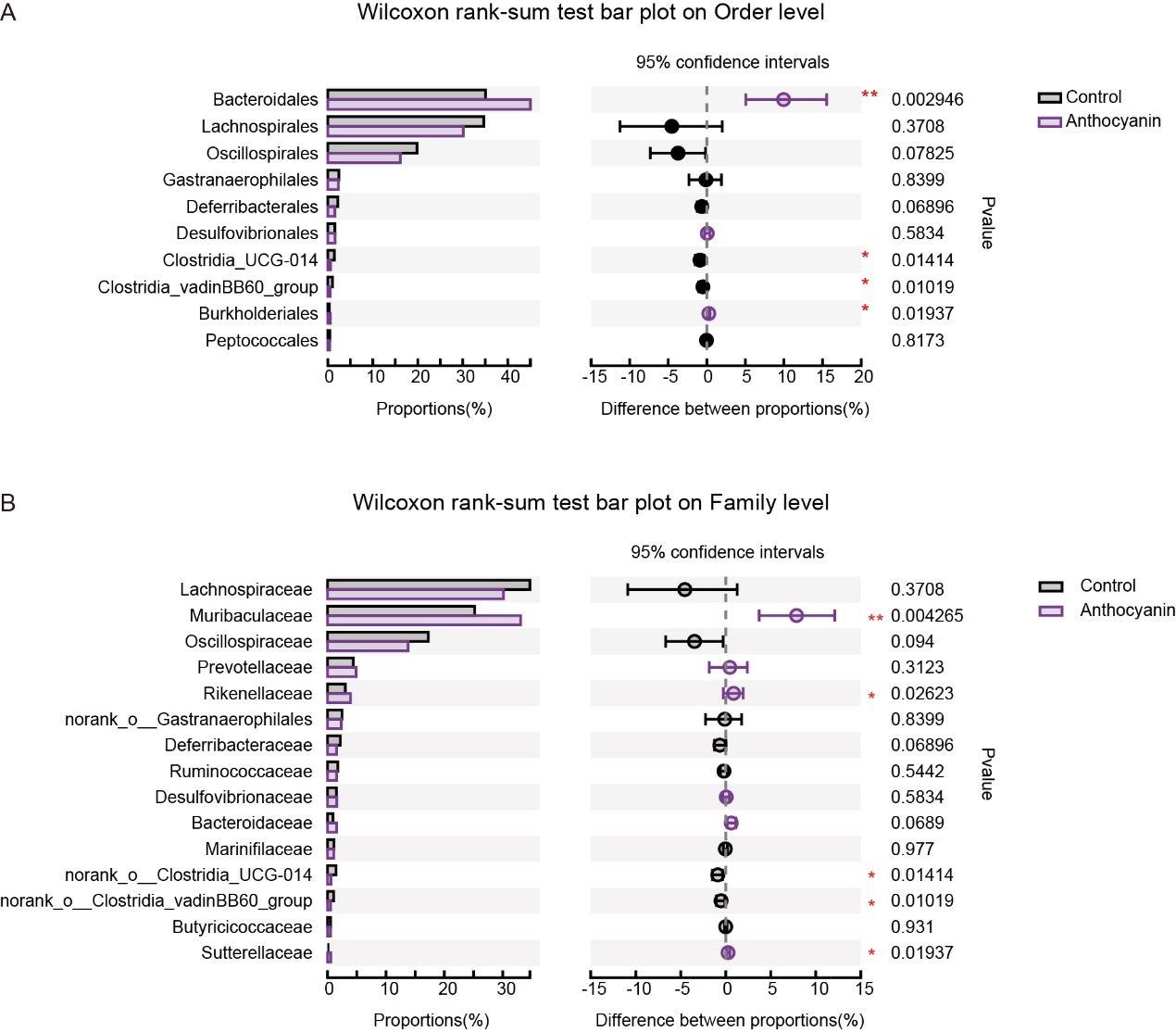


**Supplementary Figure S8.** **B****ar plots of Wilcoxon rank-sum test at the order (A) and family (B) level.** Data are means ± SEM. * denotes *p* < 0.05, ** denotes *p* < 0.01.


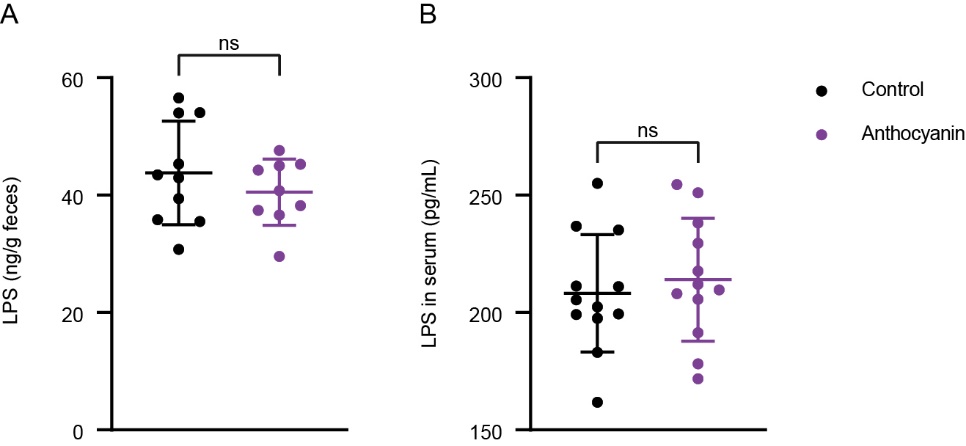


**Supplementary Figure S9.** **The LPS levels in feces (A) and serum (B) after two-week anthocyanin treatment.** Data are means ± SEM. Student’s *t*-test.


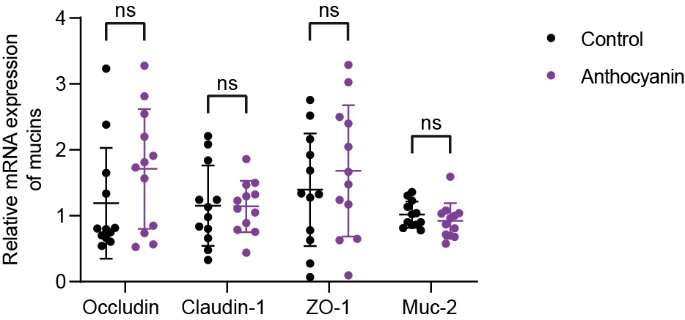


**Supplementary Figure S10.** **The relative mRNA expression of mucins after two-week anthocyanin treatment was expressed as fold changes against β-actin.** Data are means ± SEM. Student’s *t*-test.

**
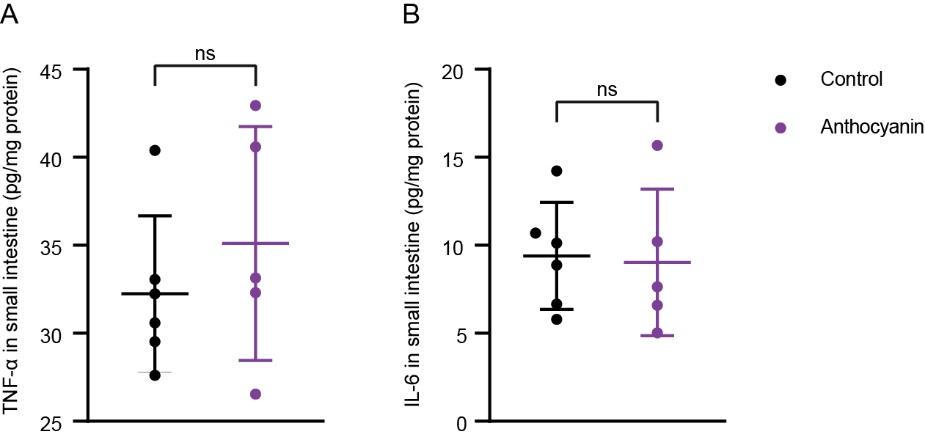
**

**Supplementary Figure S11.** **The effect of anthocyanins on the levels of TNF-α (A) and IL-6 (B) in small intestine.** Samples were collected after two-week anthocyanin treatment. Data are means ± SEM. Student’s *t*-test.


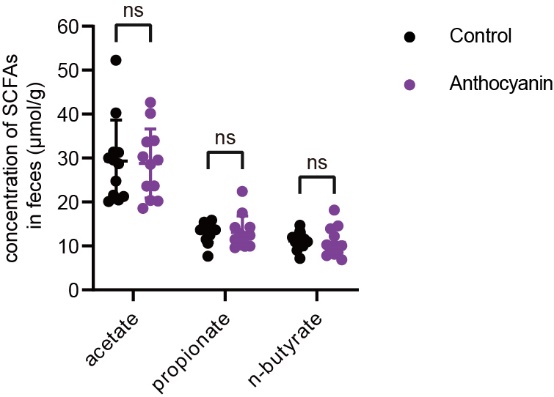


**Supplementary Figure S12. Quantification of the concentrations of acetate, propionate and *n*-butyrate in feces by gas chromatography.** Data are means ± SEM. Student’s *t*-test.

## Supplementary Table

**Supplementary Table S1. Antibody list for Flow cytometry.**

| **Name** | **Host** | **Clone** | **Company** | **Dilution** | **Cat#** |
| --- | --- | --- | --- | --- | --- |
| Anti-mouse CD45.2 APC | Mouse | clone 104 | eBioscience | 1:100(FC) | 17-0454-82 |
| Anti-mouse CD45R FITC | Mouse | clone RA3-6B2 | eBioscience | 1:100(FC) | 11-0452-82 |
| Anti-mouse IgA PE | Mouse | clone mA-6E1 | eBioscience | 1:100(FC) | 12-4204-83 |

**Supplementary Table S2.** **Primers used for quantitative RT-PCR detection**

| **Gene** | **Forward primer 5’-3’** | **Reverse primer 5’-3’** |
| --- | --- | --- |
| Claudin-1(1) | TCCTTGCTGAATCTGAACA | AGCCATCCACATCTTCTG |
| Occludin(1) | ACTCCTCCAATGGACAAGTG | CCCCACCTGTCGTGTAGTCT |
| ZO-1(1) | CCACCTCTGTCCAGCTCTTC | CACCGGAGTGATGGTTTTCT |
| Muc-2(1) | GATGGCACCTACCTCGTTGT | GTCCTGGCACTTGTTGGAAT |
| Ang4(2) | CTCTGGCTCAGAATGTAAGGTACGA | GAAATCTTTAAAGGCTCGGTACCC |
| Cryptdin-1(2) | TCAAGAGGCTGCAAAGGAAGAGAAC | TGGTCTCCATGTTCAGCGACAGC |
| pLys(2) | GCCAAGGTCTACAATCGTTGTGAGTTG | CAGTCAGCCAGCTTGACACCACG |
| β-defensin1(3) | CGCATTCTCACAAGTCTTGGACGAAC | TGCTCTTACAACAGTTGGGCTTATCTGT |
| RegIIIγ(2) | CCTCAGGACATCTTGTGTCTGTGCTC | TCCACCTCTGTTGGGTTCATAGCC |
| ß-actin(1) | GCTGAGAGGGAAATCGTGCGTG | CCAGGGAGGAAGAGGATGCGG |

Abbreviation: ZO-1 means zonula occludens-1; Muc-2 means mucin 2; Ang4 means angiogenin 4; plys means lysozyme; RegIII-γ means regenerating islet-derived protein 3-gamma.

**Reference**

1. Volynets V, Rings A, Bárdos G, Ostaff MJ, Wehkamp J, Bischoff SC. Intestinal barrier analysis by assessment of mucins, tight junctions, and α-defensins in healthy C57BL/6J and BALB/cJ mice. *Tissue Barriers* (2016) 4(3):e1208468. doi: 10.1080/21688370.2016.1208468.

2. Al Nabhani Z, Lepage P, Mauny P, Montcuquet N, Roy M, Le Roux K, et al. Nod2 Deficiency Leads to a Specific and Transmissible Mucosa-associated Microbial Dysbiosis Which Is Independent of the Mucosal Barrier Defect. *J Crohns Colitis* (2016) 10(12):1428-36. doi: 10.1093/ecco-jcc/jjw095.

3. Williams H, Crompton RA, Thomason HA, Campbell L, Singh G, McBain AJ, et al. Cutaneous Nod2 Expression Regulates the Skin Microbiome and Wound Healing in a Murine Model. *Journal of Investigative Dermatology* (2017) 137(11):2427-36. doi: 10.1016/j.jid.2017.05.029.
